# Comparing the precision of two digital PCR applications for copy number comparisons in protists

**DOI:** 10.1038/s41598-025-13143-8

**Published:** 2025-07-25

**Authors:** Megan Gross, Thorsten Stoeck, Quentin Mauvisseau, Audun Schrøder-Nielsen, Micah Dunthorn

**Affiliations:** 1 Department of Ecology, Rheinland-Pfälzische Technische Universität Kaiserslautern-Landau, Kaiserslautern, Germany; 2 Natural History Museum, University of Oslo, Oslo, Norway

**Keywords:** Protists, ndPCR, ddPCR, Copy number, Precision, Reproducibility, Biological techniques, Ecology, Microbiology, Molecular biology

## Abstract

Microorganisms play key roles in ecosystem functioning, making reliable methods for assessing their dynamics essential. Advances in molecular technologies now enable their quantification in environmental samples based on DNA marker genes. Among these, digital PCR has emerged as a powerful tool for detecting and quantifying organisms based on their gene copies, with numerous platforms that are currently available. However, these platforms differ in their underlying technologies, and comparative studies that evaluate the performance and reproducibility remain limited. Here we compared different platform parameters across the QX200 digital droplet PCR from Bio-Rad and the QIAcuity One nanoplate-based digital PCR from QIAGEN. We used synthetic oligonucleotides and DNA extracted from varying cell numbers of the ciliate *Paramecium tetraurelia* and additionally tested the impact of two restriction enzymes on gene copy number quantification. Both platforms demonstrated similar detection and quantification limits and yielded high precision across most analyses. We found a general tendency of higher precision using the HaeIII restriction enzyme instead of EcoRI, especially for the QX200 system. Gene copy number estimates from ciliate DNA were reproducible between platforms and showed a linear trend for an increasing number of cells for both platforms. These findings highlight the importance of cross-platform evaluations to ensure robust and reproducible gene copy number analysis in unicellular eukaryotes and support a potential broader application of digital PCR in environmental monitoring studies.

**Supplementary Information:**

The online version contains supplementary material available at 10.1038/s41598-025-13143-8.

## Introduction

Measuring the temporal and spatial distributions of organisms is important for understanding the functioning and stability of environments^1,2^. Reliable tools therefore need to be chosen to increase the ability of detecting the organisms and to ensure a true reflection of the abundance of those organisms^3,4^. Numerous molecular methods have been developed to do this from environmental samples^5–7^. Digital PCR (dPCR) is currently considered to have great potential as it enables the absolute quantification of nucleic acids^8^, and numerous studies have already successfully applied it in aquatic environments to detect or quantify bacteria^9–12^, microbial eukaryotes^12–17^, as well as macro-organisms^18–23^. However, comparisons of different digital PCR platforms in their ability to accurately or precisely estimate gene copy numbers are scarce^24–28^.

The concept of dPCR to estimate absolute gene copy numbers was first developed in the 1990’s^29,30^, and over the past 30 years, continuous progress was made towards the development of robust dPCR methodologies. In dPCR, absolute gene copy numbers are quantified and then used for abundance estimations of the intended target species^31–33^. Contrary to quantitative PCR (qPCR) the estimation of gene copy numbers is not carried out in a bulk reaction. Instead, the reaction mix is separated into several thousands of reactions in which the PCR then takes place^34,35^. Because DNA molecules are randomly distributed during the dPCR process, each reaction either carries no (negative reaction) or one up to a few DNA molecules (positive reaction). After end-point PCR, fluorescence signal detection is performed on each reaction to evaluate the number of DNA molecules. This signal detection can be based on double-stranded DNA binding dyes (e.g. EvaGreen) or on oligonucleotide probes that have been labelled with a fluorescent dye and bind to the gene of interest^36,37^. Using Poisson statistics, the absolute number of gene copies per reaction can then be determined^38^.

Different types of dPCR technologies are now commercially available, including microwell chip-based, droplet-based, and nanoplate-based platforms^38–40^. Although they are all based on single-molecule PCR and use Poisson statistics, they rely on different mechanisms to perform the partitioning and therefore fluorescence readings. For example, the droplet-based systems generate droplets to partition their PCR reactions and after end-point PCR, droplets are scanned with a laser to detect fluorescent signals^41^. Microwell chip-based or nanoplate-based platforms perform the partitioning by separating their PCR reactions into thousands of nanoscale chambers^38^. Positive and negative partitions are afterwards determined via imaging of the nanoplate. Despite their different approaches, all platforms aim to increase the sensitivity of the measurement and ultimately want to achieve highly accurate and precise copy number estimations. Since dPCR is often seen as an improved technology to qPCR, researchers have mainly compared the performance of these two technologies with each other. These studies showed that dPCR is the better choice for applications that require a higher precision^42^ and is less susceptible to inhibition caused by humic acids in environmental samples^43^. Studies also found that despite their similar dynamic range, dPCR is more sensitive and can detect gene copy numbers that occur in low abundances^43,44^. However, only limited research has been done comparing the performance across different digital PCR platforms using the same DNA material^24,26,45^.

Although strategies have been formulated and incorporated into a comprehensive guideline for the application of digital PCR (dMIQE)^46^, with the aim to minimize error and standardize analysis steps, this theoretical framework does not substitute for an actual evaluation of the performance across different platforms. Comparing different platforms with respect to parameters such as precision, accuracy, sensitivity or repeatability is vital for the interpretation and validation of the produced data. This is especially important for evaluating and comparing the abundances of microbial eukaryotes across different studies. Single-celled organisms including diatoms, dinoflagellates and ciliates can show copy numbers ranging between a few thousand up to half a million copies, in addition to intraspecific variations^32,47–51^. Some of the evaluated genes can also occur in tandem repeats that may influence their accessibility^52^. Transferring the conclusions from studies that already compared the performance of different platforms with each other is therefore problematic since they mainly used known plasmid reference material. To compare the results from different studies using different platforms, it must be ensured that the precision and accuracy of the different platforms is in the same range, even for organisms with high gene copy numbers. Ciliates are particularly suitable for this type of comparison as they naturally show a great variability in gene copy numbers and many species have already been studied extensively, allowing for a better comparison^32,50,51^.

In this study, we compared the performance of the QX200 droplet digital PCR (ddPCR) platform from Bio-Rad (Temse, Belgium) with the QIAcuity One nanoplate digital PCR (ndPCR) from QIAGEN (Hilden, Germany) using DNA extracted from increasing cell numbers of the model ciliate *Paramecium tetraurelia* (*P. tetraurelia*) as well as from synthetic oligonucleotides. We evaluated: (1) the Limit of Detection (LOD) and Limit of Quantification (LOQ) as a measure of sensitivity, (2) the precision and accuracy of both platforms, and (3) the level of agreement between copy number estimations of *P. tetraurelia* cells. We also tested two different restriction enzymes to evaluate their influence on the accessibility of tandemly repeated genes. The findings of this study demonstrate the tradeoffs associated with different digital PCR platforms, and highlights the necessity of such methodological comparisons.

## Results

### Limit of detection (LOD) and limit of quantification (LOQ)

Measurements for the DNA starting concentrations of synthetic oligonucleotides showed deviations from the concentrations provided by the manufacturer (measured concentration: 1.68 ng/µL; concentration provided by manufacturer: 2.5 ng/µL). For the other dilution levels, DNA measurements were not possible due to the limits of the fluorometer and were therefore not compared. The evaluation for the dynamic range of both digital PCR platforms showed interpretable results for six of 11 dilution levels, ranging from < 0.5 copies/µL input to > 3000 copies/µL input. For some of the dilutions (1.68 ng/µL, 0.168 ng/µL, 0.0168 ng/µL, 0.00168 ng/µL), the concentration oversaturated both the ndPCR and ddPCR platforms and therefore had to be excluded from further analysis. In addition, the lowest dilution level (1.68E^−10^ ng/µL) was excluded for both platforms because the signal was too weak to be differentiated from background noise. The remaining six dilution samples were used to estimate the LOD and LOQ for both platforms. The LOD of ndPCR was ca. 0.39 copies/µL input (15.60 copies/reaction for a 40µL reaction; see Data S1.1 in Supplementary Material) and the LOD of ddPCR was ca. 0.17 copies/µL input (3.31 copies/reaction for a 20µL reaction; see Data S1.2 in Supplementary Material).

**Fig. 1:**
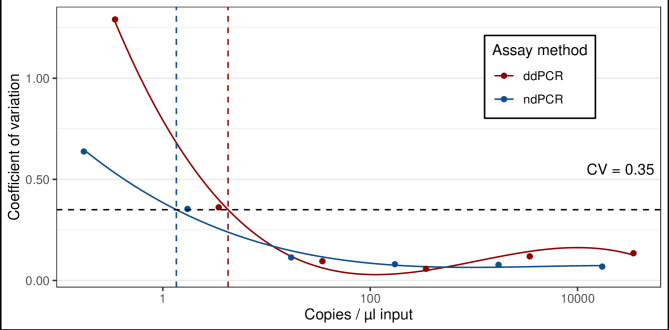
Limit of quantification (LOQ) for dPCR (blue) and ddPCR (red). The dashed black horizontal line represents the 0.35 coefficient of variation (CV) that was used to calculate the LOQ. Dashed blue vertical line represents the LOQ for dPCR (1.35) and dashed red vertical line represents LOQ of ddPCR (4.26).

The LOQ was determined using the best model-fit for both platforms. Based on the AIC value, the 3rd degree polynomial model gave the best fit for both platforms. For ndPCR the LOQ was determined at 1.35 copies/µL input (54 copies/reaction) and for ddPCR the LOQ was determined at 4.26 copies/µL input (85.2 copies/reaction). LOQ and best model fit are shown in Fig. 1.

### Accuracy and precision

**Fig. 2:**
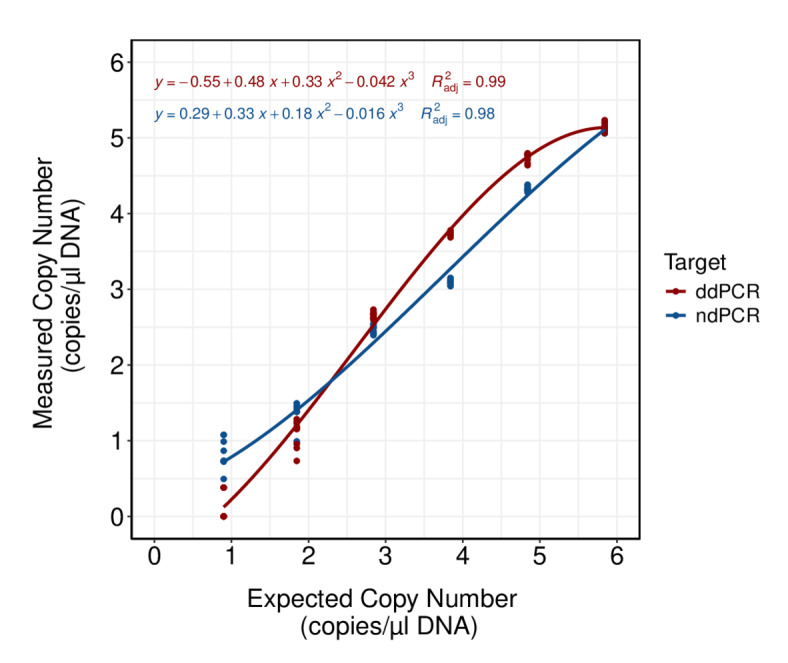
Relationship between log-transformed expected gene copy number per µl DNA calculated from Eq. 1 and log-transformed measured gene copy number per µL DNA resulting from digital PCR experiments. Red data points and regression line indicates ddPCR results and blue indicates dPCR results. For both dataset a 3rd degree polynomial model was fitted.

Comparison of expected against measured gene copy number of synthetic oligonucleotides showed good model fit for both platforms (ndPCR: R^2^_adj_ = 0.98, ddPCR: R^2^_adj_ = 0.99; Fig. 2). Despite the high correlation coefficients, all measured gene copy numbers were consistently lower than the expected gene copies for both platforms, with ddPCR showing a slightly better agreement than ndPCR. This effect was especially pronounced for ddPCR at both ends of the dynamic range and for ndPCR with increasing concentrations. Highest accuracy was achieved for mid-concentration dilution levels for ddPCR and for the two lowest dilution levels for ndPCR. Coefficient of variation (CV) for both platforms indicated precise results for all dilution groups above the LOQ thresholds with CVs ranging between 7 and 11% for ndPCR and 6 to 13% for ddPCR (Fig. 1). The highest precision for ddPCR was achieved for concentrations of ca. 270 copies/µL input. For ndPCR the precision was highest for ca. 3000 copies/µL input but achieved similar precision for concentrations between ca. 31–534 copies/µL input (CV 8%).

**Fig. 3:**
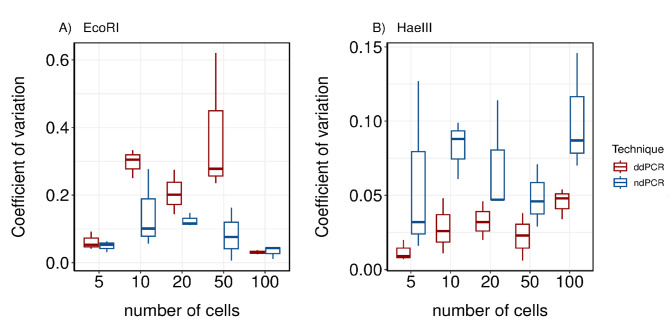
Boxplots showing coefficient of variation of technical replicates for dPCR (blue) and ddPCR (red) for different cell numbers of *P. tetraurelia* using (A) EcoRI or (B) HaeIII as a restriction enzyme in the assays.

Precision estimates using DNA originating from *P. tetraurelia* showed differences depending on the choice of restriction enzyme (Fig. 3). CVs were higher for ddPCR compared to ndPCR for all cell numbers except 100 cells for the assay using EcoRI. For ddPCR, CV-values using EcoRI varied between 2.5% and 62.1% depending on the cell numbers with the highest value for one of the 50 cell samples. When using HaeIII as a restriction enzyme, the overall precision was increased for ddPCR with all CVs lower than 5% (Fig. 3B). For ndPCR, restriction enzyme choice had less effect on the overall precision with CV-values ranging between 0.6% and 27.7% for EcoRI assay (Fig. 3A) and 1.6% and 14.6% for the HaeIII assay (Fig. 3B). On average, %CV values were also slightly lower for ndPCR using the HaeIII restriction enzyme.

### Platform performance and reproducibility

Analyses of gene copy numbers for varying cell numbers of *P. tetraurelia* ranging from 5 cells to 100 cells showed quantifiable results for all samples on both platforms (see Data S1.3 in Supplementary Material). All cell numbers showed significantly higher mean estimated gene copy numbers for ndPCR compared to ddPCR for both assays using either EcoRI or HaeIII as restriction enzyme (Wilcoxon signed-rank test: W = 1015, *p* < 0.001; W = 943, *p* < 0.001, respectively). Average gene copy number increased proportionally with cell numbers for both platforms and restriction enzymes. Correlation coefficient was highest for ndPCR using EcoRI (R^2^ = 0.95, *p* < 0.001) followed by ddPCR using HaeIII (R^2^ = 0.94, *p* < 0.001), ddPCR using EcoRI (R^2^ = 0.93, *p* < 0.001) and ndPCR using HaeIII (R^2^ = 0.92, *p* < 0.001). Despite the general increase in gene copy number with cell numbers, we found one exception. For both platforms and assays, the average gene copy number of 5 cells was higher than the average gene copy number of 10 cells (see Data S1.3 in Supplementary Material).

Comparisons between biological replicates of each cell number showed CV-values ranging between 12 and 42% (EcoRI) and between 14 and 44% (HaeIII). The lowest variation between biological replicates for both platforms was observed for the samples of 100 cells using EcoRI restriction enzyme with 11.77% for ddPCR and 13.03% for ndPCR. The highest variation was found for samples of 20 cells with CV of 44.41% using ndPCR and HaeIII and 41.63% using ddPCR and EcoRI (see Data S1.3 in Supplementary Material). The choice of restriction enzyme had greater influence on %CV of biological replicates when ndPCR was used with overall higher values for all cell numbers when using HaeIII (see Data S1.3 in Supplementary Material).

**Fig. 4:**
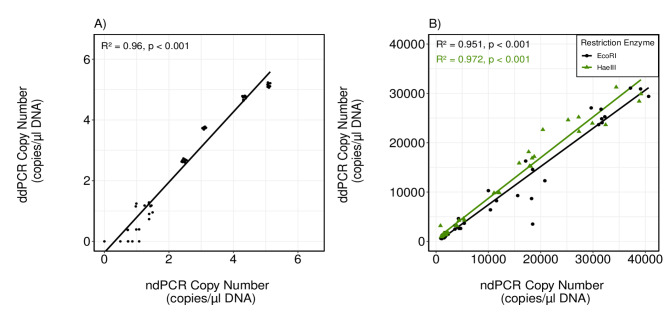
**(A)** Linear regression between dPCR and ddPCR for log-transformed copy number comparison of a 10-fold dilution series. (**B**) Linear regression between dPCR and ddPCR for copy number comparison of different cell numbers of *P. tetraurelia* separated by restriction enzyme.

To compare the reproducibility across both platforms, gene copy numbers obtained from synthetic oligonucleotides as well as *P. tetraurelia* DNA were used. Linear regression between both platforms for the 10-fold dilutions of synthetic oligonucleotides showed a good linear relationship with higher variability at lower concentrations than at mid or higher concentrations (R^2^ = 0.96; Fig. 4A). However, paired t-tests showed significant deviations for copy number estimations between ndPCR and ddPCR for five dilution levels except for the second lowest dilution averaging around 69.16 copies/µL DNA (see Data S1.4 in Supplementary Material). Because log-transformation could not reduce heteroscedasticity a weighted Bland-Altman approach was chosen to evaluate the differences between platforms in more detail. Weighted Bland-Altman analysis showed a negative bias for ndPCR compared to ddPCR measurements suggesting on average lower values for ndPCR compared to ddPCR (β_0_= -0.495, p < 0.001; see Data S1.5 in Supplementary Material). The linear regression model suggested that this relative difference is consistent across the measurement range with no evidence for proportional bias (β_1_= 0.022, p > 0.05)

To test whether the choice of restriction enzyme influenced the general level of agreement between both platforms, a linear regression was performed for each restriction enzyme individually. Both regressions showed a good linear relationship between ndPCR and ddPCR with an R^2^ of 0.97 for HaeIII and an R^2^ of 0.95 for EcoRI (Fig. 4B). For the evaluation of the level of agreement between both methods using ciliate DNA, data was log-transformed to stabilize variance and avoid heteroscedasticity. Concordance Correlation Coefficient (CCC) for EcoRI restriction enzyme resulted in a moderate agreement with a value of 0.92 (95% CI: 0.88–0.95) and for HaeIII CCC resulted in a strong agreement with a value of 0.96 (95% CI: 0.94–0.98). Since CCC only accounts for the overall agreement between the two methods a Bland-Altman analysis was performed to evaluate the level of agreement between both platforms for each comparison individually and to identify potential systematic biases. Bland-Altman plots showed a positive bias between log-transformed ndPCR and ddPCR measurements, suggesting that ndPCR tends to give slightly higher values than ddPCR (see Data S1.6 in Supplementary Material). However, these biases were not statistically significant for EcoRI or HaeIII (β_0_= 0.23, p > 0.05; β_0_= -0.08, p > 0.05; respectively). For HaeIII the mean difference was closer to zero than EcoRI indicating almost no systematic bias. In addition, the limit of agreement range was narrower for HaeIII, and most points clustered tightly around the mean difference indicating high precision. Both Bland-Altman plots showed no obvious outliers except one point below − 0.5 (HaeIII; see Data S1.6B in Supplementary Material) and one point above 0.7 (EcoRI; see Data S1.6 A in Supplementary Material). Further, the linear regression model of the log-transformed data suggested no proportional bias for EcoRI (β_1_= -0.02, p > 0.05) or for HaeIII data (β_1_= 0.034, p > 0.05) indicating consistent differences across all concentration levels.

## Discussion

This study highlights the importance for comparing the performances and reproducibility across different digital PCR platforms for gene-copy-number analyses of single-celled eukaryotes. First, we observed a similar dynamic range and found high sensitivity for both the nanoplate-based dPCR from QIAGEN and the droplet-digital PCR from Bio-Rad. Second, we showed that precision of both platforms increased using a 4-cutter restriction enzyme and that precision was in general higher using the respective enzyme and ddPCR. Third, gene copy number analyses of synthetic oligonucleotides and different cell numbers of *P. tetraurelia* showed that reproducibility across both platforms was strongly dependent on input DNA concentrations. Together these main results show that using the optimal concentration range allowed high precision and reproducibility across digital PCR platforms for gene copy number analyses of ciliates.

Our findings suggest that although both platforms are highly sensitive and can detect DNA concentrations below 0.5 copies/µL, ddPCR resulted in a lower LOD compared to ndPCR. However, when evaluating the LOQ we found lower values for ndPCR. Since we applied the same assay and samples for both platforms, these differences most likely arise from general technical differences, causing a certain degree of measurement uncertainty that can influence the precision of estimates especially at the lower range of copy number concentrations^42,53,54^. Indeed, similar results have been observed in another study who found that optimal LOD and LOQ varied between platforms when using the same assay^24^. This measurement uncertainty could be due to pipetting errors or variations in droplet or partition volume, which have a strong influence on platform sensitivity^53,55,56^. For ndPCR, a plate-specific volume-precision factor is incorporated into the Software Suite that accounts for slight size-differences of the nano-chambers to increase precision between technical replicates and could have improved LOQ measurements. It is also possible to perform multiple imaging steps, in which the exposure time and gain (signal amplification factor) can be changed. By contrast, with ddPCR, fluorescence detection can only be performed once by scanning with a laser beam. Performing multiple imaging steps with minor adjustments could potentially improve detection and quantification limits, especially for weak signals. Additionally, the concentration of each point of the dilution series can impact the estimation of the LOD and LOQ, and while we used a 1:10 dilution series, using a 1:5 dilution would have increased the number of dilution points and likely led to a higher resolution when estimating the LOD and LOQ. This finer resolution would have allowed for a more precise identification where the detection levels drop below a reliable threshold. For environmental studies that want to develop an assay to quantify ciliates or other protists, including potential pathogens, a lower limit of quantification could be preferred for low abundant and/or rare species. However, since both platforms showed high analytical sensitivity with similar LOD and LOQ estimates, we consider both technologies to be suitable for the aforementioned.

Only a few studies have used digital PCR so far for the quantification of gene copy numbers in ciliates, especially with regards to potential environmental applications^16,32,57^. Here, we obtained LODs and LOQs on both ndPCR and ddPCR platforms within a similar range as observed in these previous studies. Although the LOD and LOQ estimates produced in this study refers to what could be achieved when analysing optimal samples (i.e. samples without non targeted DNA or other co-extracted substances), it is unsure how these estimates would be impacted when analysing environmental samples that are often influenced by extraction efficiencies or inhibition due to the presence of e.g. humic acids^4^. However, several studies have already demonstrated the advantages of dPCR for quantifying low- abundance targets in complex environmental samples^22,58,59^. Unlike qPCR, which processes the entire sample in a single bulk reaction, dPCR partitions the sample into thousands of individual reactions, effectively diluting inhibitors and reducing their impact^59,60^. Moreover, because dPCR enables absolute quantification, it removes the need for standard curves which can be difficult to generate and apply accurately in complex environmental samples^61,62^. To further address potential issues related to low extraction efficiency or inhibition effects, internal positive controls of known concentration can be added prior to DNA extraction^58^. These controls can serve as correction factors, helping to improve the accuracy of quantitative results.

Digital PCR platforms have demonstrated strong potential for quantifying ciliates in environmental samples^32^. For both platforms and restriction enzymes tested, we found strong correlations between average gene copy numbers and increasing cell number. The only deviation from this trend was found when analyzing low cell concentration (i.e. 5 and 10 cells), where no significant increase was observed. Ciliates, which often have several thousand copies of the same gene, can exhibit substantial intraspecific variations in gene copy number^32,50,51,63^. This variation can complicate the differentiation especially in samples with closely spaced cell numbers^49,64^. This was further evident in our comparison of biological replicates, which showed coefficients of variation ranging from 12% to 44%, depending on the restriction enzyme used. Additionally, the use of restriction enzymes was able to decrease results variation, suggesting that further testing should be performed in future studies to increase results reliability. These findings suggest that low concentration samples may fall within the variability range of the assay, and thus represent the lower limit for reliably differentiating *P. tetraurelia* cell counts using this approach. Despite its limitations, dPCR represents a valuable addition to the suite of available quantification methods for protists and has already successfully been applied within the environment^13,16^. Traditionally, ciliate abundances have been estimated through microscopy techniques, which often provide more accurate estimates than molecular approaches^65^. However, they remain time-consuming, require considerable taxonomic expertise, and are often constrained by limited sampling volumes^65^. Digital PCR techniques offer a promising alternative by enabling the estimation of ciliate abundances through average gene copy numbers per cell, which can then be applied to environmental samples. Furthermore, dPCR allows for the detection of low-abundance taxa that may remain undetected through microscopy and offers the potential to distinguish cryptic species that are morphologically indistinguishable. Even if absolute quantification across all ciliate taxa remains challenging due to variable gene copy numbers and unknown calibration values, dPCR still reliably reveals spatial and temporal distribution patterns. In similar environmental studies on protists, dPCR has been successfully used to track community shifts^13,15^ or to monitor harmful species^16,17^, despite limitations in biomass estimation. Digital PCR, together with other emerging approaches, such as low-coverage genome skimming^66^ or multispecies genotyping by sequencing^67,68^, could therefore play a key role in advancing the molecular quantification of protists in complex environmental samples.

For both platforms examined here, accuracy estimations showed issues using the synthetic oligonucleotides. Although accuracy was dependent on the concentration levels, discrepancies showed variations of more than 50% of the expected concentration for some of the dilution levels, suggesting a systematic bias affecting accuracy of both platforms. Because DNA concentrations of the quantifiable dilution levels were too low to be measured with the fluorometer, deviations could result from inaccurate DNA starting concentrations used for calculating the expected gene copy number. Since gene copy numbers varied by tenfold (see Fig. 2) we considered errors caused by inaccurate dilutions to be minor. Estimating gene copy numbers requires optimal threshold setting to avoid a misclassification of partitions. Any misclassification could result in type I (false positive signal) or type II (false negative signal) errors that can influence precision and accuracy estimations^69^. Although we acknowledge that some of the partitions could have been misclassified causing a type II error and resulting in lower copy numbers than expected, this error would be minor since no partitions of intermediate fluorescence intensity were observed for ndPCR or ddPCR. Taking all other factors into account, we cannot exclude the possibility that the deviations from the expected gene copy numbers are caused by degraded or damaged synthetic oligonucleotides. Despite these possible explanations, the discrepancies we found in this study remain inconclusive and results differentiate from the literature that in general found high accuracy for digital PCR using plasmid DNA for their assays^26^. Since accuracy is important for the enumeration of ciliates and other protists based on DNA sequences^70^, future studies need to address the efficiency of the platforms in more detail, especially before any environmental applications. Studies showed that the use of internal positive controls can help to reduce bias by standardizing the variations caused by factors such as DNA extraction efficiency or amplification efficiency^19,71,72^. Using internal positive controls as a correction factor together with optimized DNA concentrations will be necessary to increase accuracy, especially for future environmental studies.

We further assessed the precision of both platforms and examined the potential influence of two commercially available restriction enzymes on gene copy number quantification. Since rDNA genes occur in tandem repeats and can have several thousands of copies in ciliates, it is important to fragment and linearize input DNA to make genes more accessible^52^. This fragmentation can improve platform precision and resolution by reducing random error, ultimately resulting in increased gene copy number estimates^46^. We compared the 4-cutter restriction enzyme HaeIII with the 5-cutter restriction enzyme EcoRI. Although the choice of restriction enzyme did not significantly influence the gene copy numbers, we did observe a general tendency of ddPCR precision being more affected by this. Other studies have observed similar effects for ddPCR with a positive influence on amplification efficiencies when linearizing plasmid DNA^26^. For ddPCR, genomic DNA with high molecular weight is known to influence the average droplet volume, making restriction enzyme choice important for improving precision of the platform^73^. By contrast, the nanoplate based digital PCR system seemed to be less affected by the choice of restriction enzyme. For both platforms, using HaeIII resulted in higher gene copy numbers for most of the samples and increased the precision of both platforms. Other studies that investigated the impact of restriction enzyme choice on the number of amplifiable targets using ddPCR found contrasting results, with overall less positive events for assays using restriction enzymes that produce smaller fragments^17,27^. They stated that while restriction enzymes can enhance gene accessibility, certain types may cause mechanical damage or unintended fragmentation, potentially reducing gene copy numbers. Prior to conducting any experiments, we tested both restriction enzymes to confirm their cutting sites and ensured they did not cleave within the target fragment. We attribute our differing results to the higher cutting frequency of HaeIII, which has a shorter recognition sequence compared to EcoRI. This increased frequency may have enhanced the accessibility of our gene of interest. These findings highlight the importance of evaluating different restriction enzymes as their properties may influence platform precision.

Many studies have reported the importance of assessing qualitative measures of the performance of different digital PCR platforms especially with a focus of comparing them to quantitative PCR^24^. We found that gene copy number analyses showed systematic but predictable differences across the two digital PCR platforms, suggesting a general agreement between both measurement methods, aside from some outliers. Using organismal DNA of ciliates showed the highest reproducibility between both platforms, especially when using HaeIII as restriction enzyme and for concentrations between 100 and 1000 copies/µL of reaction. As shown elsewhere^56,69,74^ DNA input concentrations strongly influence the precision and reliability of gene copy numbers and negatively affect the agreement between different platforms. For future studies, optimal concentration ranges need to be addressed carefully to allow comparisons between different studies. Considering all investigations for *P. tetraurelia* DNA we found a general underestimation of ddPCR copy numbers compared to ndPCR. As mentioned earlier, restriction enzyme digestion had a greater effect on ddPCR assay and could have resulted in lower overall gene copy numbers compared to ndPCR. Comparisons between the QX200 ddPCR and the QIAcuity ndPCR platforms were so far mainly conducted for medical investigations of viruses or mutation analyses^25,45^. These studies showed a general agreement between both platforms and suggest that the observed differences might be caused by threshold settings. Although we cannot neglect a potential effect of threshold settings on variations between both platforms, we assume this effect to be minor. To date there are no comparisons between platforms using highly variable ciliate DNA. Other studies that compared reproducibility and general platform performance have used DNA originating from plasmids, tumor cells, bacteria or viruses^25,26,28,45,73^. The results obtained in this study further showed the great potential of digital PCR technologies. This is highlighted by the finding that despite the target gene occurring in tandem repeats, good agreement between platforms was achieved at certain concentration levels.

Comparing sensitivity and reproducibility across different digital PCR platforms is important to evaluate potential limitations and general performance metrics^75^. However, there are also general platform-specific characteristics that were not addressed in this study that could be relevant for future research endeavors. For example, a study that aims to detect rare or low copy number targets might be interested in the maximum template input volume which strongly depends on the platform and can reach from 11µL (ddPCR QX200) to 22 µL (ndPCR QIAcuity 26k plate)^76,77^. Similarly, studies that want to use digital PCR for absolute abundance estimations of ciliates (or other high copy number protists) first need to evaluate the gene copy numbers per single cell and their intraspecific variations. As was shown in^32^, single cell analyses require high DNA template input volumes (20 µL) to quantify gene copies. For some ciliates with high single cell gene copy numbers this could also be conducted with ddPCR despite the lower maximum template volume. However, for species for which lower gene copy numbers are assumed, higher template input volumes might be necessary. There are extensive reviews discussing different digital PCR platforms that include further information on the general principles and costs that were not addressed in this study^38,77^. Deciding which platform to choose therefore depends on the research question. In this study we showed that despite potential differences, both platforms showed a good agreement and were able to produce precise results for a wide dynamic range. Their high throughput and sensitivity could allow future monitoring of different protists groups including environmental key species as well as indicator species.

## Materials and methods

### Samples and DNA extraction

Here, we used DNA originating from *P. tetraurelia* stock 51 cells as well as synthetic oligonucleotides. Synthetic oligonucleotides covered a 237 bp fragment of the large-subunit (LSU) of the rRNA gene of *P. tetraurelia* (see Data S1.7 in Supplementary Material), and were provided as gBlocks Gene Fragment by Integrated DNA Technologies (IDT, Coralville, Iowa, USA). For *P. tetraurelia*, samples containing DNA from 5 up to 100 cells were used in this study. Part of these DNA samples came from^32^, including the samples containing 5, 50 and 100 cells. We additionally extracted DNA from 10 to 20 cells for this study. These cells originated from the same cultures as the previous data but were extracted at a later time point.

A detailed description of culture maintenance and DNA extraction can be found in^32^. Briefly, three *P. tetraurelia* cultures were set up in parallel in 50mL culture flasks to allow for true biological replicates. From each culture a subsample was taken and living cells were then transferred into small drops of sterile Volvic^®^ using finely drawn glass Pasteur pipettes and a stereo microscope with a 45x magnification (Olympus SZ60). Each cell was then washed five times to remove extracellular DNA prior to DNA extraction with the DNeasy Blood & Tissue Kit (QIAGEN) following the manufacturer’s instruction. This was applied to all cells and DNA was eluted using AE buffer in a final volume of 100 µl. Extracted DNA samples were then stored at −20 °C before further processing.

### Molecular analysis

Nanoplate digital PCR (ndPCR) and droplet digital PCR (ddPCR) analyses were carried out in two separate laboratories. ndPCR analyses were performed at the RPTU in Kaiserslautern while the ddPCR analyses were performed at the Natural History Museum in Oslo. Dilution series of synthetic rRNA oligonucleotides (a total of 11 dilution levels at 1:10) were used to determine the Limit of Detection (LOD) and Limit of Quantification (LOQ) of the assays used in this study, and to compare the precision of both platforms. Samples originating from living *P. tetraurelia* cells were used to compare reproducibility of copy number analyses across different platforms and to test their suitability to quantify ciliates. In addition, for the samples with varying cell numbers, we also tested if the choice of restriction enzyme influenced the performance of the platforms for copy number estimations. We selected two restriction enzymes from New England Biolabs (NEB, Frankfurt am Main, Germany), one 6-cutter restriction enzyme (EcoRI) and one 4-cutter restriction enzyme (HaeIII), that were suggested for the QIAcuity EvaGreen dPCR assay. For quantifying the gene copy numbers, we used the PAC-F/R (5’ -CAAGAGTCGGGTTGTTTGGG-3’/5’ -GCCCTATGAAGTACCATTACCG-3’) primers from^32^ that amplify a 237 bp long fragment spanning across the LSU rRNA gene.

### Nanoplate digital PCR (ndPCR)

Copy number analyses were performed on the nanoplate-based QIAcuity One Digital PCR System (QIAGEN) using the automated workflow provided by the QIAcuity Software Suite v.2.1. The system performs all steps in one machine including partitioning, amplification of the target gene with end-point PCR and fluorescent detection of gene copy numbers using an automated imaging step. All samples were processed under a PCR-workbench that was treated with UV-light 30 min prior to the handling of samples. In addition, all equipment was cleaned with 80% ethanol before being used under the PCR-workbench. The PCR-reaction was set up according to the manufacturer’s recommendations using the EvaGreen^®^ assay. For all the analyses, 24-well nanoplates with 26,000 partitions were chosen to maximize the number of possible reactions. The PCR reaction consisted of 13.3 µL QIAcuity EvaGreen^®^ Mastermix, 4 µl forward and reverse primer (0.4 µM), 0.5 µL EcoRI or 1 µL HaeIII restriction enzyme (0.25 U µL^−1^), 1 or 2 µL of DNA depending on the experiment and nuclease-free water up to a final reaction volume of 40 µL. We used 2 µL of input DNA for the experiments with varying cell numbers and 1 µL of input DNA for experiments with synthetic oligonucleotides. We repeated the analyses for the dilution series with 10 replicates for each of the 11 dilution levels, and all samples extracted from *P. tetraurelia* cells were analyzed in triplicate. Reaction mix and DNA of each sample were first prepared in standard end-point PCR tubes (Brand, Life Sciences, Carl Roth, Karlsruhe, Germany) and were afterwards transferred into the nanoplate wells. Nanoplates were then sealed and run on the QIAcuity One platform with the following reaction conditions: 2 min at 95 °C, followed by 40 cycles of 15 s denaturation at 95 °C, 15 s annealing at 60 °C and 15 s extension at 72 °C and a final cool down for 5 min at 40 °C. On each nanoplate, 3 non-template controls and a positive control were included. For the non-template controls, nuclease-free water was used and for the positive control DNA extracted from a pure culture of *P. tetraurelia* cells was used at a concentration of 3 ng µL^−1^. Following amplification, imaging of the nanoplates was performed using the green channel to detect the EvaGreen^®^ fluorophore and setting the exposure duration to 250 ms with a gain (signal amplification factor) of 3. To increase the precision of the measured sample, the Software Suite additionally provides a Volume Precision Factor (VPF). By applying the VPF, potential variations in partition sizes are taken into account and the exact cycled volume of each well is specified before the calculation of the sample concentration (further details are provided in Chap. 7.4.6 of the QIAcuity User Manual). The relative fluorescent intensity (RFU) was set to a common threshold of 75 for all experimental runs to separate positive from negative partitions.

### Droplet digital PCR (ddPCR)

Copy number analyses for ddPCR were conducted on a QX200 ddPCR system (Bio-Rad). Before any handling of samples, the surface and equipment were treated with 5% Deconex solution and absolute ethanol to remove any contaminants. Each reaction was carried out in a final volume of 20 µL, consisting of 10 µl Bio-Rad ddPCR EvaGreen^®^ supermix, 0.5 µL forward and reverse primer (0.125 µM), 0.5 µL EcoRI or 1 µL HaeIII restriction enzyme (0.25 U µL^−1^), 1 or 2 µL of input DNA and the respective volumes of nuclease-free water to reach 20 µL reaction volume. For the dilution series experiments, 1 µL of template DNA was used and for the *P. tetraurelia* samples of varying cell numbers 2 µL was used. As for ndPCR, all samples of the dilution series were run with 10 replicates and all samples extracted from *P. tetraurelia* DNA were run in triplicates. In addition, a minimum of 3 non-template and one positive control was added to each 96-well plate (see section above for further details). Each ddPCR reaction mix was then added to a DG8™ cartridge together with 70 µL of Droplet Generation Oil for EvaGreen and placed into the QX200 Droplet Generator that generates up to 20,000 droplets. After droplet generation, a 40 µL reaction mix was transferred into 96-well plates and plates were sealed using a PX1 PCR Plate Sealer (Bio-Rad). ddPCR plates were run on Bio-Rad CFX96 Real-Time System (Bio-Rad) with the following conditions: 5 min at 95 °C, 40 cycles of 30 s denaturation at 95 °C and 1 min annealing/extension at 60 °C with a ramp rate of 2 °C/s, followed by 5 min at 4 °C, 5 min at 90 °C and hold at 8 °C. Read-out of droplets was then performed on the QX200 droplet reader (Bio-Rad) and copy numbers were calculated using the QX manager software v.2.0. To separate positive from negative events, a common fluorescence threshold was set to 18,000 for all experiments.

### Data analysis

All analysis were conducted in R v.4.3.1 using the packages tidyverse v.2.0.0^78^, scales v.1.3^79^, car v.3.1-2^80^, rstatix v.0.7.2^81^, ggpubr v.0.6.0^82^, knitr v.1.45^83^ and DescTools v.0.99.57^84^. All result figures were created using ggplot2 v.3.5.1^85^.

#### LOD and LOQ

To estimate the minimum amount of target that can reliably be detected and quantified we calculated the LOD and LOQ for ndPCR and ddPCR^86^. For both platforms, total gene copy numbers per reaction were calculated by multiplying the measured concentrations (copies/µL) by the reaction volume (40 µL for ndPCR and 20 µL for ddPCR), to reflect the absolute number of target molecules per reaction. Definitions describing both vary among studies. Here, we refer to LOD as the minimum concentration at which 95% of the positive samples are detected^87^. For the definition of LOQ we followed^86^ describing it as the lowest concentration at which technical replicates show a coefficient of variation (CV) below or equal to 35%. The DNA starting concentration to estimate the LOD and LOQ of both platforms was 1.68 ng/µL (see sections above for more detail). DNA starting concentrations were measured using the Quantus fluorometer with the QuantiFluor One dsDNA dye (Promega, Walldorf, Germany). Since the linear range only covers concentrations between 0.2 and 400 ng only the first two dilution levels could reliably be quantified. The LOD was calculated for each platform using the R script from^44^ and the LOQ was calculated based on the percent CV threshold as in^88^.

#### Reproducibility, precision and accuracy

Precision and accuracy of both platforms was tested using the results originating from the dilution series. First, the expected copy number per µL DNA was calculated based on the equation:

1 $$Number~of~copies~\left( {molecules} \right)=~\frac{{X~ng~*~6.0221*{{10}^{23}}\frac{{molecules}}{{mol}}}}{{\left( {146,288.9\frac{g}{{mol}}*1*{{10}^9}\frac{{ng}}{g}} \right)}}$$

Where X is the amount of amplicon (ng), 6.022*10^23^ represents the Avogadro’s constant, 146,288.9 g/mol is the molecular weight of our fragment and 1*10^9^ is the conversion factor. A linear regression was performed using the Akaike’s information criterion (AIC) to determine the best model fit.

Reproducibility was tested by comparing the linear relationship between copy number estimations of both platforms. Copy numbers resulting from synthetic oligonucleotides as well as copy numbers from different *P. tetraurelia* cell numbers were used for evaluating reproducibility. First, difference in estimated gene copy number across platforms was tested using an appropriate paired test statistic (Paired Student’s t-test or Wilcoxon Signed-Rank test) based on the normality and homogeneity of variance criteria. To evaluate the level of agreement between ndPCR and ddPCR, different complementary statistical methods were applied including Concordance Correlation Coefficient (CCC), the regression coefficients and Bland-Altman analysis. CCC evaluates both precision (Pearson correlation) and accuracy (closeness to a 1:1 agreement line) when quantifying the level of agreement between methods and is defined as:

2 $$CCC=~\frac{{2\rho {\sigma _x}{\sigma _y}}}{{\sigma _{x}^{2}+~\sigma _{y}^{2}+{{\left( {{\mu _x} - ~{\mu _y}} \right)}^2}}}$$

where is the Pearson correlation coefficient, are the standard deviations, and are the means of ndPCR and ddPCR measurements, respectively. Bootstrapped 95% confidence intervals were calculated to assess the stability of agreement estimates. Bland-Altman was used to assess the systematic bias and limits of agreement between both platforms and to visualize differences based on concentration levels. If log-transformation was not able to account for heteroscedasticity (variability of differences increases with higher copy number values), a weighted approach was used and compared with the traditional approach to evaluate robustness. Weights were determined using inverse-variance weighting, where the weight assigned to each data point was calculated as:

3 $${w_i}=~\frac{1}{{\sigma _{i}^{2}}}$$

where represents the estimated variance of differences for each measurement. Using this approach ensures that measurements with lower variability contribute more to the estimation of bias and limits of agreement. The weighted mean difference (bias) is calculated as:

4 $${\mu _w}=~\frac{{\sum {w_i}~ \cdot ~{d_i}}}{{\sum {w_i}}}$$

where is the difference between ndPCR and ddPCR for each sample. To further evaluate the precision of both platforms the coefficient of variation (CV) was calculated for the technical replicates of all cell number samples.

The ndPCR and ddPCR data as well as the R code for all figures and analyses can be found here: : 10.5281/zenodo.15829164.

## Supplementary Information

Below is the link to the electronic supplementary material.

Supplementary Material 1

## Data Availability

Data and code are available online: https://doi.org/10.5281/zenodo.15829164.
