# Supplementary material for: Comparing the precision of two digital PCR applications for copy number comparisons in protists
Source: Sci Rep. 2025 Jul 25;15:27095. doi: 10.1038/s41598-025-13143-8 (PMC12297429; doi:10.1038/s41598-025-13143-8)
Supplement: Supplementary file 1 — Supplementary Material 1 [file 41598_2025_13143_MOESM1_ESM.pdf]

Supplemental Information for:

Comparing the precision of two digital PCR applications for copy number comparisons in protists

Megan Gross[1*], Thorsten Stoeck[1], Quentin Mauvisseau[2], Audun Schrøder-Nielsen[2], Micah Dunthorn[2]

[1]Department of Ecology, Rheinland-Pfälzische Technische Universität Kaiserslautern-Landau, Kaiserslautern, Germany

[2]Natural History Museum, University of Oslo, Oslo, Norway

*Corresponding author: gross.megan@rptu.de

Table of Contents:

**S1.1** Limit of detection (LOD) for ndPCR	Page 2
**S1.2** Limit of detection (LOD) for ddPCR	Page 3
**S1.3** Average gene copy number per µL *P. tetraurelia* DNA	Page 4
**S1.4** Statistical comparison between ndPCR and ddPCR	Page 5
**S1.5** Weighted Bland-Altman for dilution comparison between platforms	Page 6
**S1.6** Bland-Altman for *P. tetraurelia* DNA comparison between platforms	Page 7
**S1.7** Sequence of synthetic oligonucleotides	Page 8

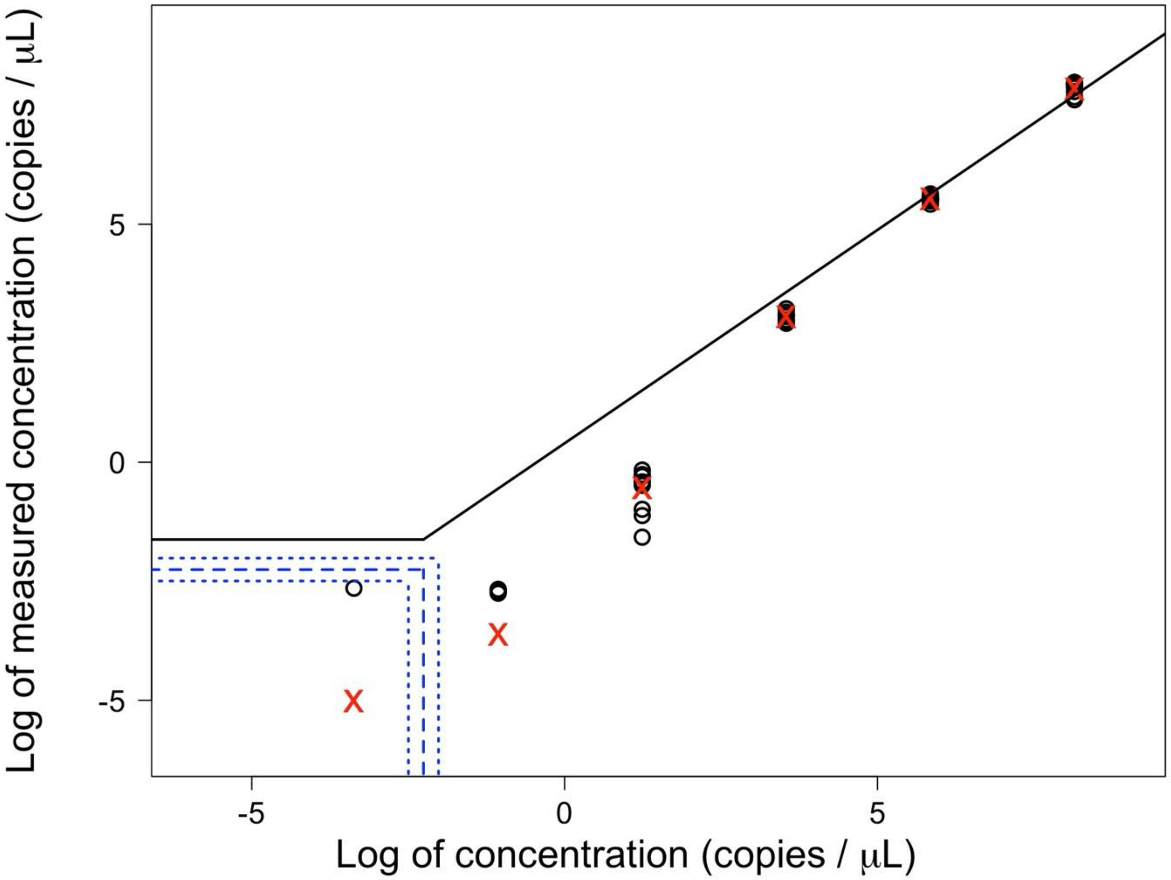

S1.1: Estimated limit of detection (LOD) for ndPCR, based on a 1:10 serial dilution of synthetic oligonucleotides across six concentration points with 10 replicates each. Individual replicate measurements are shown as black circles; red Xs denote the mean measured concentration at each dilution level. The LOD is defined as the point of intersection between the linear regression fitted to the quantifiable concentrations (solid black line) and the background response plateau where the instrument fails to distinguish signal from noise (dashed blue line, with upper and lower 95% confidence bounds shown as dotted blue lines). The estimated LOD was 0.3975086 copies/μL. An intersection is not visible in the plot because the regression and plateau curves intersect outside the range of measured concentrations.

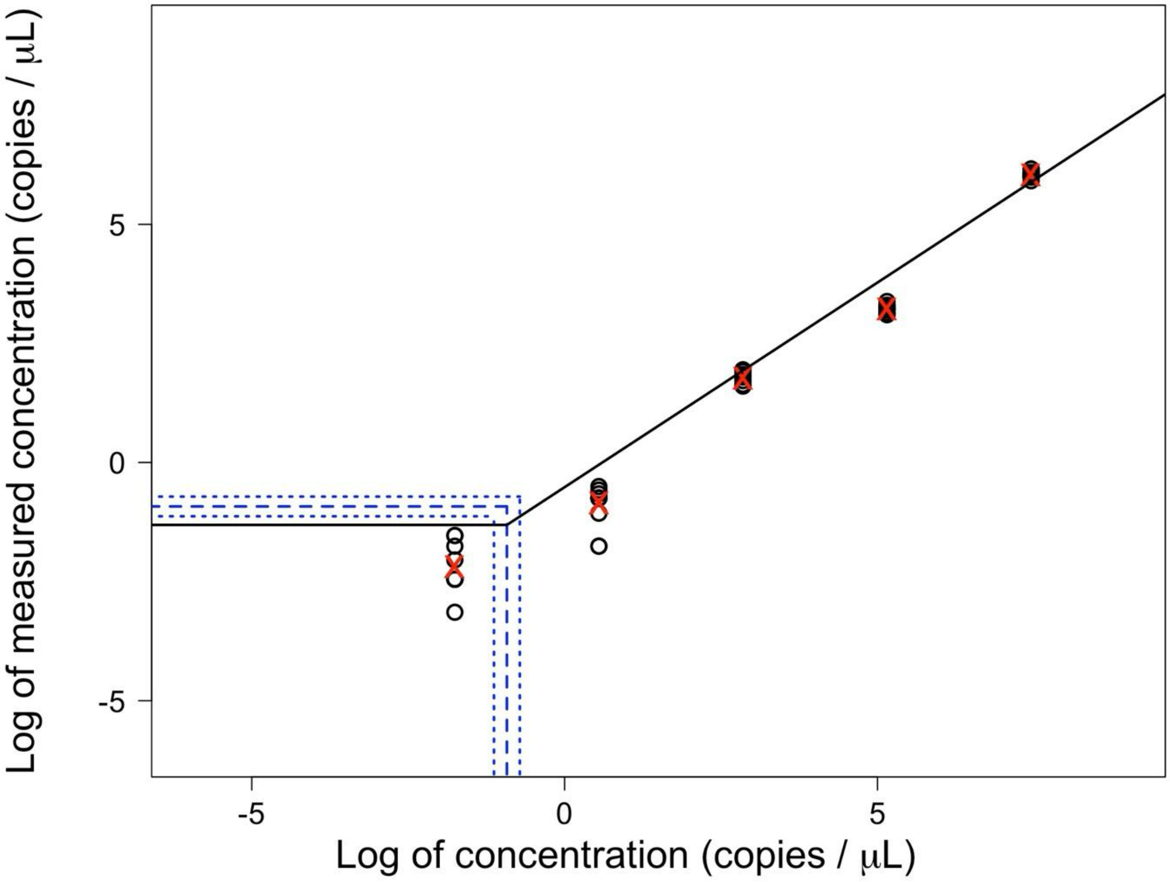

S1.2: Estimated limit of detection (LOD) for ddPCR, based on a 1:10 serial dilution of synthetic oligonucleotides across five concentration points with 10 replicates each. Individual replicate measurements are shown as black circles; red Xs denote the mean measured concentration at each dilution level. The LOD is defined as the point of intersection between the linear regression fitted to the quantifiable concentrations (solid black line) and the background response plateau where the instrument fails to distinguish signal from noise (dashed blue line, with upper and lower 95% confidence bounds shown as dotted blue lines). The LOD was 0.1653171 copies/μL.

S1.3: Average gene copy number per µL DNA (GCN[1]) and corresponding coefficient of variation (CV) for different number of extracted cells of *P. tetraurelia* obtained from ndPCR and ddPCR using A) EcoRI or B) HaeIII as a restriction enzyme in the assay. CV refers to variation between biological replicates. SE = standard error.

A)

cell number	ndPCR		ddPCR	
	GCN[1] (SE)	CV (%)	GCN[1] (SE)	CV (%)
5	1671.87 (162.96)	16.58	1419.20 (194.37)	23.48
10	1372.67 (144.90)	15.99	860.51 (129.00)	18.82
20	3546.02 (843.74)	40.59	2628.91 (667.42)	41.63
50	15626.89 (2553.24)	27.67	9947.81 (1884.33)	23.47
100	33872.22 (2584.30)	13.03	27005.56 (1856.99)	11.77

B)

cell number	ndPCR		ddPCR	
	GCN[1] (SE)	CV (%)	GCN[1] (SE)	CV (%)
5	1484.89 (140.29)	15.59	1386.88 (141.58)	17.67
10	1378.40 (246.73)	30.53	1184.69 (126.36)	18.37
20	3336.07 (925.73)	44.41	3132.17 (745.34)	41.15
50	15750.22 (2070.90)	22.57	14238.2 (2265.17)	27.50
100	30552.00 (3937.43)	21.61	25759.48 (2110.09)	13.93

S1.4: Statistical comparison between ndPCR and ddPCR for each Group. Group corresponds to the 6 different 10-fold dilutions ranging from 691585.5 (D1) - 6.92 (D6) expected copies. n.s. = not significant.

Group	Test Type	Test Statistic	adj. *p value*	p. signif.
D1	Paired Welch's t-test	-2.38	0.0427	*
D2	Paired Student's t-test	-21.99	3.93E-09	***
D3	Paired Student's t-test	-55.34	1.03E-12	***
D4	Paired Student's t-test	-10.17	3.10E-06	***
D5	Paired Student's t-test	2.24	0.0519	n.s.
D6	Paired Student's t-test	6.08	0.0001	***

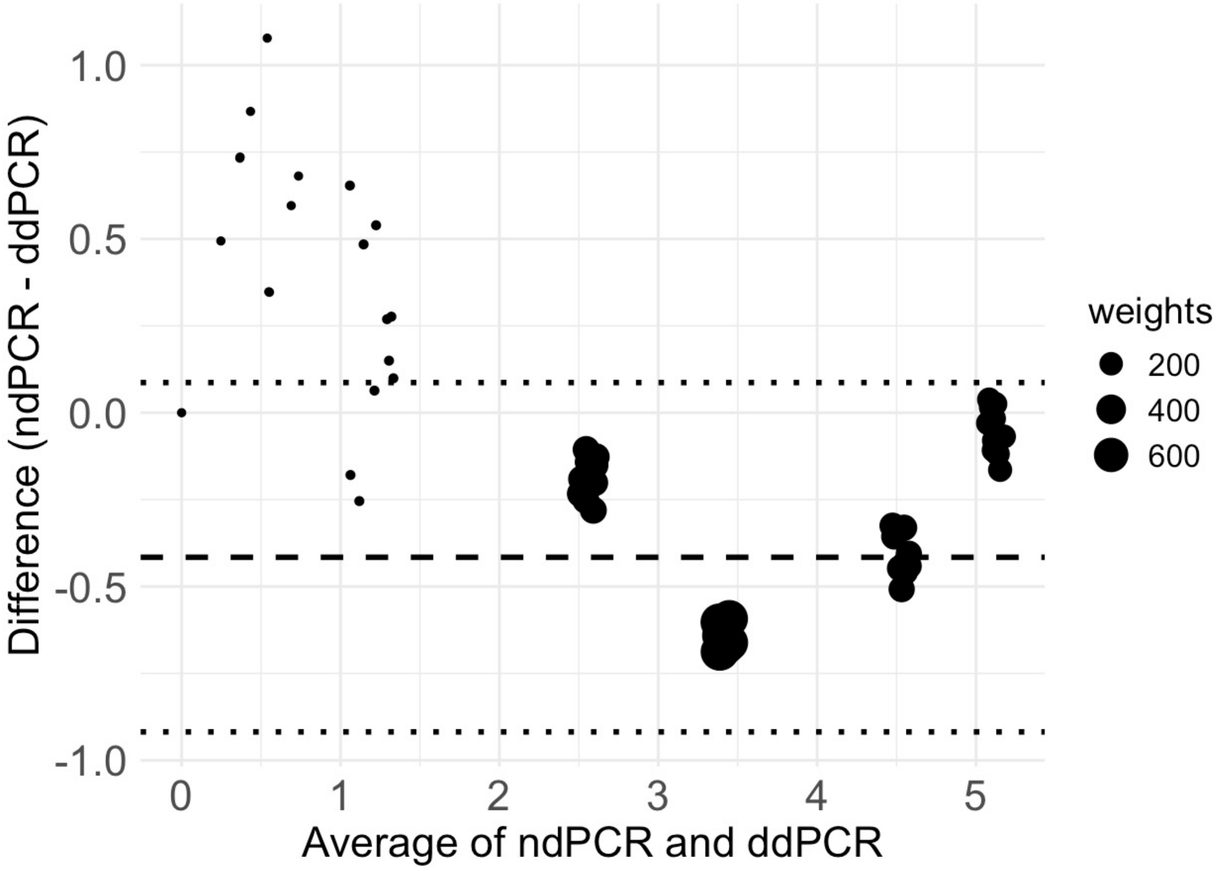

S1.5: Weighted Bland-Altman comparing agreement between ndPCR and ddPCR for log-transformed copy numbers for six dilution levels. Different weight of each point indicates different levels of reliability (larger points = greater reliability). Dashed line represents the mean bias, and dotted lines represent the 95% limits of agreement (mean difference ± 1.96 x weighted standard deviation).

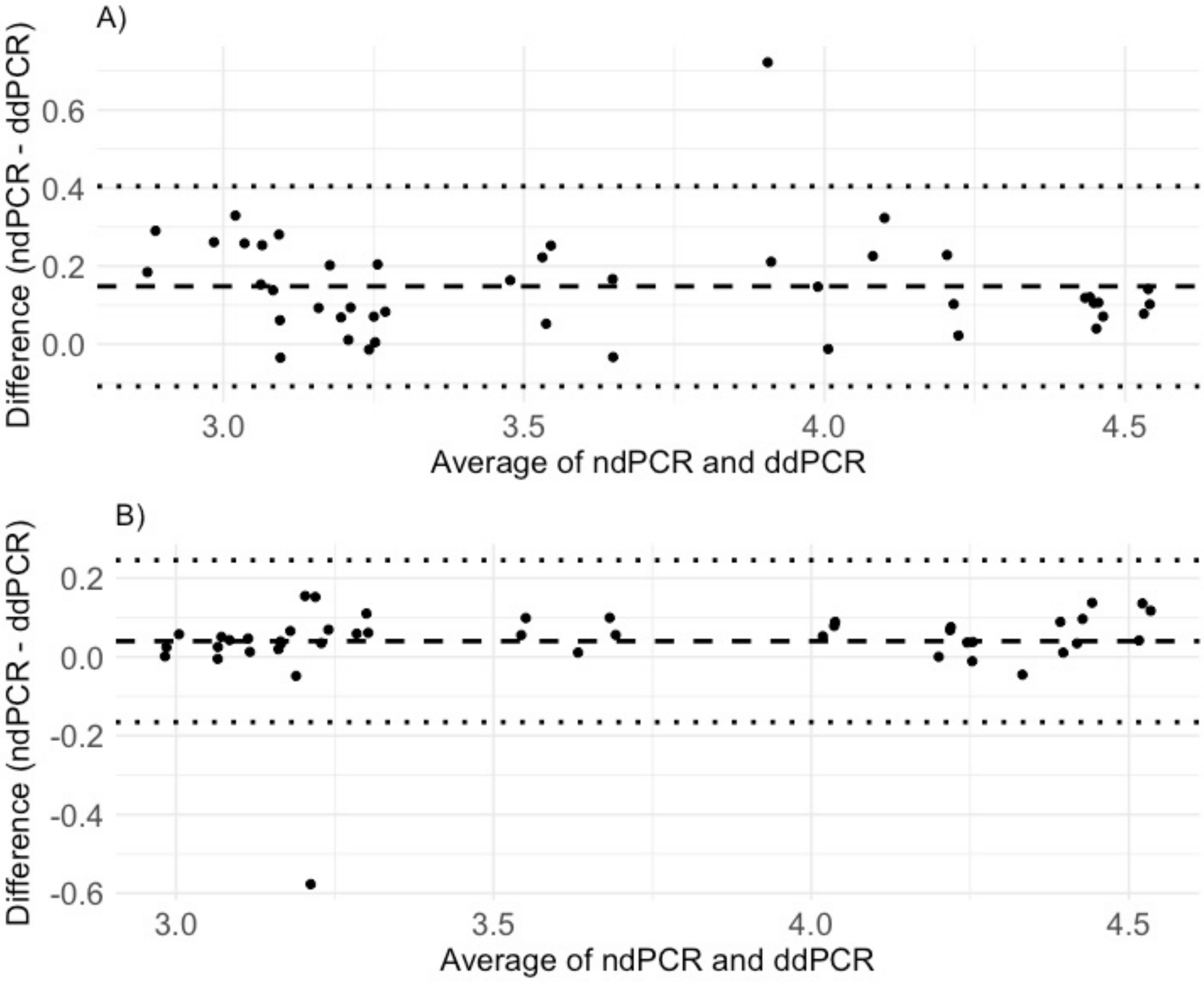

S1.6: Bland-Altman plots comparing agreement between ndPCR and ddPCR copy numbers of *P. tetraurelia* cells. Data was log-transformed to avoid heteroscedasticity. Comparisons are separated by restriction enzyme A) EcoRI and B) HaeIII. Dashed line represents the mean bias, and dotted lines represent the 95% limits of agreement (mean difference ± 1.96 x weighted standard deviation).

S1.7: Sequence of synthetic oligonucleotides

5'- CAA GAG TCG GGT TGT TTG GGA TTG CAG CCT AAA ATG GGA GAT AAA CTT CTT CTA AAG CTA AAT ATT TAT GGG AAA CCG ATA GCA AAC AAG TAC TAC GAA GGA AAG ATG AAA AGA ACT TTG AAA AGA GGG TTA AAA GAC TTG AAA TCG TTG AGG AGA AAA GCG GTA GAA GAG AAA TGA TTT CAT TTA GAA GTA TGT AGT TAT GTA GGT GTC TTC TGC GGT AAT GGT ACT TCA TAG GGC– 3'
